# InterConnect: A European project that is changing the way energy is consumed

**DOI:** 10.12688/openreseurope.15227.1

**Published:** 2023-03-01

**Authors:** Pierre Jean-Philippe

**Affiliations:** 1 SHS (Humanities and Social Sciences), ISEN (Higher Institute of Electronics and Digital Technology) Yncréa Méditerranée, Toulon, France

**Keywords:** Energy, ENR, Consumer, Economy, Ownership, Connected objects

## Abstract

Project InterConnect is a major European project focusing on energy consumption. With 25 sites in Europe and more than 3,500 users, the InterConnect project has a dual economic and educational benefit for users, which should lead to responsible and sustainable behaviour. Fully meeting the needs of the moment and the choices of the future in terms of energy consumption and management is in line with the ambitious objectives of the European Union set out in the Paris Agreement of December 2015. The originality of this project lies mainly in the choice not to create innovation for its own sake but rather to create innovations that make the existing equipment (heaters, hot water tanks, etc.) more modern and more economical. In a context of economic and social crisis, this approach is bound to be met with a favorable response from low-income households or consumers who are also the most frequent users of energy-consuming equipment. This article is an opportunity, at the beginning of the analysis phase of the data collected during the InterConnect project, to highlight the pedagogical virtues and the capacity of such a project to influence behaviour.

## Disclaimer

The views expressed in this article are those of the author(s). Publication in Open Research Europe does not imply endorsement of the European Commission.

## Introduction

The European InterConnect project, coordinated by INESC TEC (Instituto de Engenharia de Sistemas e Computadores, Tecnologia e Ciencia), started in October 2019, should help democratize efficient energy management. France, Germany, Belgium, Italy, Portugal, Greece, and the Netherlands have mobilized 50 universities, associations, and industrial partners to implement control solutions that will make it possible to reduce the electricity consumption of households and that of public and industrial players. This ambitious and innovative experiment has been deployed at 25 sites in Europe and involves more than 3,500 users. From Greece to the Netherlands and from Portugal to Germany, connected solutions are estimated to enable users to reduce their electricity consumption by nearly 15% ^1^ through intelligent control that takes into account both the comfort needs and the specific climatic conditions of each user.

Although the main aim of this project is to reduce energy consumption, behaviour transformation is no less critical. Indeed, this experiment highlights the fun and educational aspect of applications for monitoring electrical consumption.

The news of the last few months highlights the vital need to change our energy model. The probable passing of the peak oil, the war in Ukraine, and global warming ( Devika Rao) ended the idea of unlimited growth. Europeans are now faced with scenarios they thought were science fiction. The prospect of a winter of 2022–2023, during which some will have to make supply choices, will encourage most to rationalize their electricity consumption. This European project deserves special attention for several reasons:

- In a context of solid tension in the energy market, InterConnect is a significant project in terms of the volume of the experimentation and the diversity of the means implemented. Innovation and technology play a significant role in significantly improving energy use.
- Thanks to the effectiveness of the tools and methods deployed, it is entirely conceivable to take advantage of the expected results of InterConnect to put in place an influence strategy that will make it possible to encourage end consumers, whether private, public or industrial, to change their behaviour in terms of energy use.
- Once consumers are made aware of this influencing strategy, they become real consumers (consum’acteur ( Gordon *et al.*, 2022)) of the energy market, both individually and, above all, collectively. InterConnect is, therefore, a lever for the creation of energy communities.

InterConnect intends to contribute to the democratization of efficient energy management through a flexible and interoperable ecosystem where flexibility on the demand side can be firmly integrated with practical benefits for the end users.

In fact, in the last few years, several projects, (home automation, connected heating controllers, water heating controllers, etc.), and technology providers have proposed solutions based on home automation technologies, which allow each energy user to know and control their devices. Few of these offerings were interoperable, and this limitation has always been a significant problem.

Indeed, end-users, whether private, public, or industrial, should be able to choose and change their energy supplier or service without having to replace their installations. This would allow them to adopt sustainable behaviour in terms of energy use while benefiting from technological progress.

At the same time, there has been a substantial shift towards using digital technology in the energy sector. The generalization of tools has made it possible to focus actions on the user and their behaviour in the market.

The opening up to competition between energy suppliers, encouraged by the European Union's regulatory environment, has helped improve the fluidity of all energy market sectors (consumers, suppliers, distributors, etc.) according to website totalenergies.fr in France, the electricity and gas market has been open to competition since 1 July 2007 for private individuals. Many suppliers have come to vary the market offer (Total Energie, E.Leclerc, etc.). The opening to competition started in 1999 for companies with high energy consumption (more than 100 GWh). Opening up to competition and creating a single market at the European level is part of the EU's energy policy ( energie-info). Mastering the systemic dimension has become essential. The consequences of global warming, dwindling gas, and oil reserves, and the tensions linked to the constant increase in energy needs call for a reasoned and systemic organization of consumption. This is why the InterConnect consortium brings relevant partners from all stakeholders representing this new energy paradigm.

The 50 partners include expertise in information and communication technologies (ICT), IoT ^2^, energy, data science, and software. It also consists of the entire value chain, from research and development (R&D) institutions to manufacturers, producers, and retailers. To ensure a more significant impact on a European scale, several relevant associations related to new technologies and energy are also involved. For the experimentation to reach a representative dimension, seven large-scale pilot projects in different countries and with different types of end-users are being deployed. This wide deployment ensures the credibility of the expected results.

The primary objective of these pilots is to demonstrate a true digital market environment on power systems with large amounts of intelligent systems and to reduce operating and investment costs, which will benefit energy end-users and help the EU meet its energy efficiency targets. The purpose of this article is to highlight the pedagogical virtues of such a project. This article is an opportunity, at the beginning of the analysis phase of the data collected during the InterConnect project, to highlight the pedagogical virtues and the capacity of such a project to influence encourage the consumption and use of regulation tools.

## The seven pilots

Seven pilots make up the experiment. Each one, with its own technical, societal, and geographical specificities, is implementing solutions that should eventually interconnect with those of the others. The entire InterConnect project is expected to be completed by the end of March 2024.

These solutions, which have been tested in different territories among different populations with various requirements and habits, should be able to be disseminated more widely throughout Europe and even open up new market opportunities beyond. See Table 1 and Table 2 for further information.

**Table 1.** The types of experimentation that have been or are being deployed for each pilot.

Type of equipment	
1	Connected household appliances
2	Connected modules
3	Smart meters
4	Smart heat pumps
5	Eco boilers
6	Recharging station
7	Photovoltaic panels
8	Urban heating network
9	Batteries / Storage
10	Energy management platform
11	Wind turbines
12	Customized management application

**Table 2.** The nature of the InterConnect project. See Table 1 for explanations of numbering.

Country	Location	Nature of project	1	2	3	4	5	6	7	8	9	10	11	12
Belgium
	Antwerp	Student residence	X		X									
	Gent	32 flats	X	X		X	X	X						
		New Docks + city district, consisting of 1 kindergarten, one sports facility, one administration building, and 400 flats.				X		X	X	X	X	X		
	Genk	Thor Park is a new science and business park located on a former mining site. Three buildings	X			X		X	X					
	Hasselt	Three clusters of multi-apartment buildings and approximately 70 flat units and households				X					X		X	
	Kobbegem	There are two residential buildings in the local energy community and one in the "virtual" energy community.		X	X	X		X	X		X	X		
	Oud-Heverlee	The local energy community consists of four buildings - the town hall, the OCMW office, the police station, and a crèche.		X	X	X		X	X		X	X		
	Zellik	Large-scale living laboratory to prove the concept of IoT-based technologies	X	X				X	X		X	X		X
France	Toulon	280 households, one public building, and 1 school in the TPM agglomeration	X	X	X	X		X				X		X
Germany	Norderstedt	Residential demonstration of the German pilot project	X	X		X		X	X			X		X
	Hamburg	Hotel owners and operators			X		X	X				X		
Greece	Thessaloniki Volos Athens	Installation of 200 HERON customer homes and transformation of 70 homes into smart homes.	X	X	X							X		X
Italy	Milan	Social landlord with over 600 flats	X									X		X
Netherlands	Eindhoven	The most intelligent house in the Netherlands is available to end users.	X	X	X	X	X					X		X
		Industrial building equipped with gateways, sensors and devices, actuators, and smart plugs.		X	X							X		X
Portugal	North	The residential demonstration is deployed in 250 homes in 5 cities in northern Portugal.										X		X
	The entire territory	The commercial demonstration is being developed and installed in 12 retail shops throughout Portugal, where ~75% will have local energy.						X				X		X

## InterConnect uses edge computing and IoT to optimize energy and launch new services

The awareness of the limits of fossil fuels, the revolution caused by global targets for a drastic reduction in CO _2_ emissions, and the significant spread of energy produced from renewable sources are prompting the development of distributed generation systems, the gradual space of electric mobility and storage systems, and the need to control and reduce final consumption.

The many operators who have already started to use the latest technologies to offer new products and services to their customers and align their business models with the new context have demonstrated the actual effectiveness of these technologies ( novelec.fr). However, the abundance of tools and services limits their impact on a larger scale ( la-fabrique.fr) Indeed, the lack of a common standard forces users to renew their equipment each time they move to a new home, which has often become incompatible with their new situation. It is, therefore, necessary to devise solutions to integrate all these tools to optimize energy consumption.

The Internet of Things (IoT) and edge computing are increasingly important in the evolution of product and service offerings. InterConnect builds on the inherent nature of IoT solutions, which allows the integration of a broad and heterogeneous range of technologies such as sensors, smart meters, machines (appliances, air conditioners, etc.), charging units, transformers, and inverters. Each pilot develops its own network(s) controlled by an interoperability platform intended to interconnect with the other pilots. The resulting network, which "speaks the same language", will be able to accommodate the connected tools of its neighbors without the end user having to change his equipment or habits. This configuration and management of microgrids, the dissemination of charging systems for electric vehicles, and the introduction of technologies for controlling and managing connected objects are just some of the new opportunities available to customers and energy suppliers.

In an increasingly constrained market and in order to meet the ever-growing demand, the interoperability platform is proving to be an irreplaceable ally in the future management of complex, heterogeneous and large-scale energy production, transmission, distribution and storage systems.

The InterConnect pilots should be able to harmonise their experiments by striving for an integrated and strongly market-oriented approach. To this end, the deployment of an integrated Distribution System Operator (DSO) framework for an interoperable energy system supports the exchange of standardised information between the different smart grid stakeholders, and integrates several components that can be used for flexibility, such as Home Energy Managements Systems (HEMS) ^3^ and Building Energy Management Systems (BEMS), storage, e-mobility, etc.

The use of existing Smart Grid projects ( smartgrid.gov) to demonstrate a range of technologies and solutions in an integrated environment should ensure effective management of the pilot projects. A specific transnational coordination working group has been set up to help pilot project managers ensure quality control, monitoring of key performance indicators and consistency with a common implementation plan. A common four-step methodology was adopted for each pilot:

Step 1 - Knowledge:

A field study identifies needs and opportunities (potential services and business models) and selects the most representative end-users possible who will test the solutions developed to improve their energy consumption patterns.

Step 2 - Definition:

This second step leads to the design and construction of demonstrators using creative problem solving facilitation, where challenges are tackled collectively so that solutions are shared.

Step 3 - Demonstration:

This validates or invalidates the technology with end-users who have had the opportunity to test it in their daily lives, over a sufficiently long period of time to verify its soundness. Ideally, this period extends over two winters.

Step 4 - Evaluation:

Finally, the objective review and evaluation of end-user feedback and experience will confirm the viability of the solution as is or after improvement.

A project of this magnitude requires adjustments in order to integrate other partners who can bring in other technologies or know-how. Open calls allow the ecosystem of actors involved to be broadened and provide opportunities for innovative SMEs and start-ups at European level.

## Presentation of the French pilot

In order to give a concrete idea of the experiments that are carried out by the different pilots, we propose to detail a little more precisely the work of the French pilot, which is a mix between the experiments of the pilots of the North (Germany, Belgium and the Netherlands) and those of the South (Greece, Italy and Portugal).

The societal impact study conducted on the whole InterConnect project and piloted by Yncréa Méditerranée is composed of two main chapters

- a quantitative study (based on a series of questionnaires)

- a qualitative study (based on targeted semi-structured interviews).

The first analyses commented on in this text are therefore based on the study of the first interviews conducted by the French pilot (data not yet available).

The French project concerns both public buildings and private rental or ownership housing, buildings or houses, spread over the territory of the Toulon Provence Méditerranée (TPM) metropolis.

For the benefit of private individuals, two partners have the task of deploying their solutions. These are ENGIE ^4^, one of the main French energy distributors, and ThermoVault ^5^. The former was to recruit 250 private testers and the latter 200, but the COVID-19 health crisis has seriously disrupted these recruitments. Initially, the two partners focused their recruitment on social landlords, hoping to reach a population that was sensitive to energy savings. According to the preliminary analyses of these landlords in the French sample (data not yet published), we find that this target group is more resistant to the project than other types of population. There are three reasons for this. Firstly, the tenants of these dwellings are often, septuagenarians (30 % of end-users already recruited) who, according to the interviews (data not yet published) conducted with them, are not inclined to change or who entrust these steps to their sons or daughters, who are more difficult to reach because they are absent, working or living far away. Other people, who benefit from support from social organisations (more than half of the respondents benefit from local social support) take care of all or part of their bills, so they do not feel concerned by a scheme designed to enable them to make savings. Finally, another significant part (50 % of respondents) of this population says that they are attached to their supplier, generally EDF (Electricité de France is the historical distributor in France), and do not wish to change. This population is wary of the proliferation of energy offers due to unfulfilled promises or even scams and prefers institutional or historical players. The advent of the crisis in Ukraine and the risks it poses to energy prices might have led one to believe that saving energy or benefiting from a secure tariff (ENGIE offers a fixed price for the next three years) would appeal to this lower income population. In fact, this is not the case, they show little interest in the international situation and do not measure the risk that this represents for their energy bills. All the data collected for the 7 pilots and the resulting impact assessment will be published in the second half of 2024.

The choice was therefore made to extend the recruitment to other sites such as city centers, residential and suburban areas, in order to reach a wider panel of testers. Communication campaigns were put in place on the illuminated panels of the municipalities of the Metropolis to reach more people. This means of communication did not improve recruitment. Finally, in order to reach a more technophile and environmentally aware population, communication campaigns were organised for members of economic clusters, entrepreneurial associations and engineering students and their families. The latter strategy has been beneficial and has significantly improved recruitment.

Two major lessons can be drawn from the difficulties of recruiting testers for a project of this type. The choice of targets for recruitment must be carefully considered, taking into account both the needs of the potential users and the context. It seems that in times of crisis, whatever it may be, the uncertainty of the situation encourages a majority of people to take no risks, to accept no changes, even if these changes may be beneficial to them. Moreover, in the French case in particular, canvassing is felt to be intrusive and negative, 80% of leaflets are thrown away without even being read, laws impose time limits on telephone canvassing. Indeed, potential customers are constantly solicited and many people say they are saturated with proposals, offers and promotions. It was noted that most of the letters sent by social landlords to their tenants to inform them of the holding of meetings in their building entrance to present InterConnect, were thrown in the bin without even being opened. The team in charge of holding the information sessions in the entrance halls of the buildings were able to see this when they found the letters informing them of the sessions intact in the wastepaper baskets. It is therefore preferable to rely on events in which they freely choose to participate (meetings of associations, co-ownerships, etc.).

**Figure 1.**
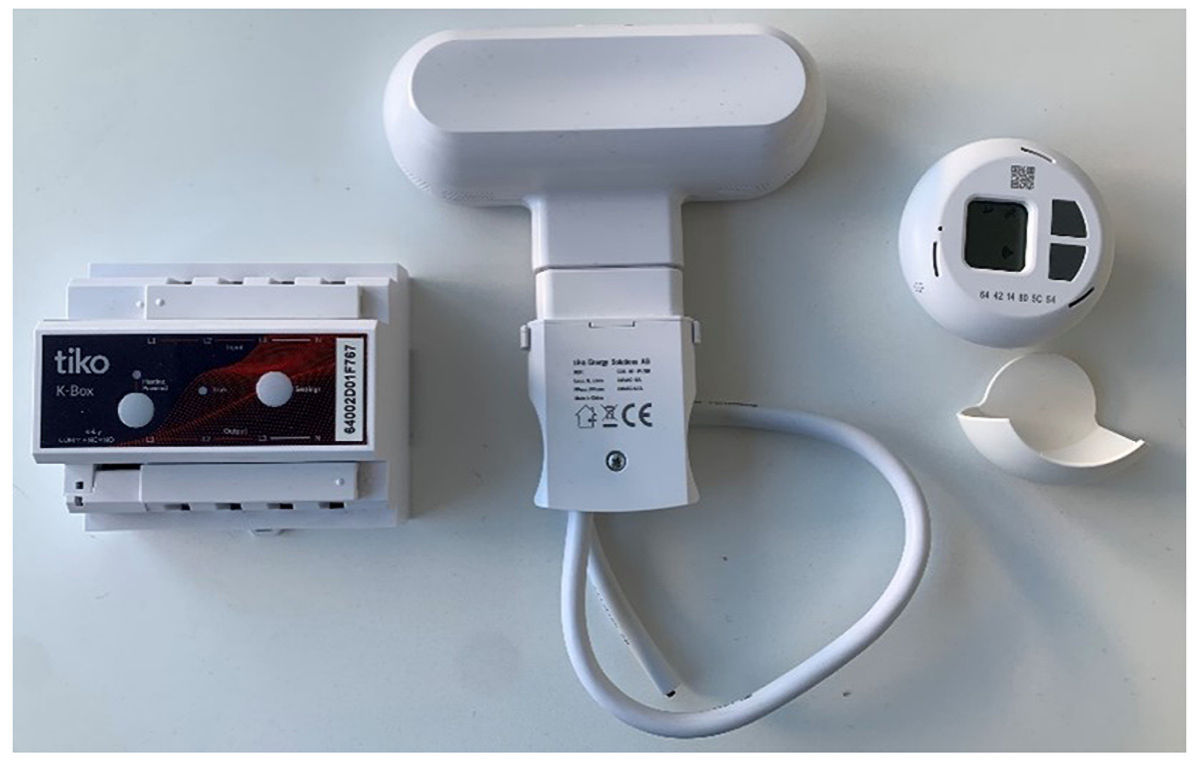
TIKO company's devices. Photo by author.

Despite recruitment difficulties, the two partners have started the deployment of their solutions on time and have kept the recruitment of testers open until the end of 2022. The choice was made to target as a priority the main areas of electricity consumption in households, namely the production of domestic hot water and heating. According to the French Agency for Ecological Transition ( ADEME) electricity consumption linked to water heating represents on average 15% of a household's energy expenditure. In this respect, the consumption of domestic hot water, used for domestic purposes, is the second largest energy expenditure item after electric heating (radiators, convectors, etc.). The two partners ENGIE and ThermoVault therefore installed in the testers' homes a system for controlling the hot water tank and up to four systems for controlling the electric heaters (TIKO boxes and probes for example – Figure 1, TIKO devices). For the sake of efficiency, only electric convector heaters and traditional hot water tanks were fitted. The latter represent 87% ^6^ and 46.5% ^7^ of the equipment respectively, which guarantees the relevance of the results obtained.

This intelligent equipment is initially used to trigger the operation of the heating or hot water tank at the time when electricity is least expensive, while guaranteeing users a level of comfort at least equal to that known before the experimentation. The control is ensured by the Smart Orchestrator platform, developed by engineers from Inetum ^8^ and ISEN ^9^ (Institut Supérieur de l'Electronique et du Numérique) YNCREA Méditerranée and the two partners, based on a dynamic tariff ^10^. While waiting for this tariff to come into force at European level, the tariff is simulated in order to calculate the real savings that would be generated. To make the experiment attractive, this simulation is carried out by the operator and the hypothetically generated savings are rewarded with gift vouchers. Thus, due to the fixed 3-year contract and the principle of reimbursing the "savings generated", the tester runs no risk of seeing his or her bill increase as a result of participating in the experiment.

In a second phase, after having familiarised themselves with the concepts of dynamic tariffs, end users can also intervene directly in the control of their heating systems by means of a digital application made available to them by ENGIE. This adaptation phase is deliberately deferred because it becomes all the more effective when the user sees the effect of the resources deployed on their bill. In this case, a user is willing to use new means that will enable him to reinforce these effects. In its May 2015 report, the Académie des technologies defines adaptation as a necessary stage of appropriation, which it defines as follows: "One of the important stages in the adoption process is that of 'instrumentation', during which the technical object must be transformed into an 'instrument', which our body, in all its components, must learn to handle. This phase requires ‘co-adaptation’, i.e. the technical object can be modified (sometimes referred to as ‘hijacking’, ranging from "customisation" to the use of a new tool). from 'customisation' to the invention of new uses, but also that our body must adopt body must adopt new behaviours, learn new gestures, develop automatisms and anticipation capacities” ( Académie des technologies). The implementation of these connected objects in the home and participation in their use require this adaptation. During discussions with end-users to present them with the mobile control application made available to them, we noted, particularly with tenants of social landlords and despite precise information, that they were not interested in the effects of the devices deployed in their homes. It is because they are invited to compare their bills that they become aware that they themselves can further improve these effects. Without this prior realisation, they generally do not take ownership of the technology.

**Figure 2.**
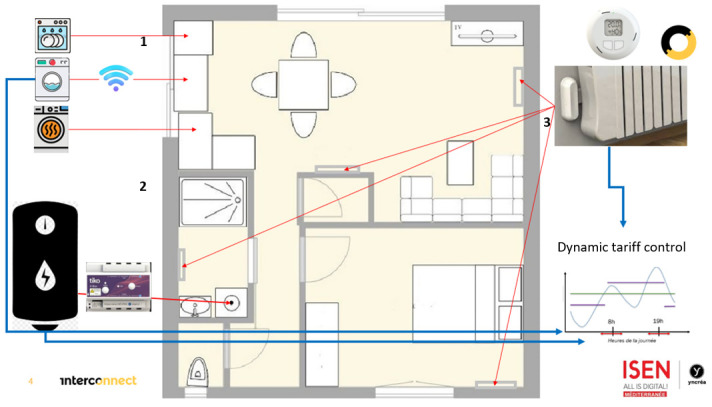
Deployment of connected objects in the framework of the InterConnect project typical home - pilot France.

Finally, to complete the range of tools that should help reduce the testers' electricity consumption, connected household appliances are also made available to them, free of charge ( Figure 2). Our partners Bosh, Whirlpool and Miele provide washing machines, tumble dryers and dishwashers for a large number of households. This provision of connected objects not only generates savings due to their more recent design, but also offers testers the possibility of triggering them either when energy is cheaper or during a peak in local renewable energy production.

The plan below shows the deployment of these facilities in a typical household.

Each eligible home is equipped with connected household appliances (1), a hot water tank control system (2) and electric radiator control systems (3). Consumption data is collected in real time using an Linky Radio Transmitter ERL set up by Enedis.

As far as public testers are concerned, two solutions are deployed. These are charging stations for electric vehicles (already operational) and collective boilers (study in progress).

Wirelane GmbH ^11^ supplies charging stations for electric vehicles. The town of Le Pradet, a commune of the Metropolis, has equipped its technical area with this equipment. The municipal team, which is very committed to the development of less energy-consuming solutions, has also had solar panels installed on the school buildings in the town centre and is planning to install car park shades equipped with solar panels.

Still within the framework of the test, public buildings in the Metropolis (museums, schools, etc.) are being identified for the installation of connected collective boilers proposed by the company Daikin, also a partner in the project. The aim here is to offer Cybergrid flexibility in the context of the overarching pilot.

Whether they are intended for private homes or public buildings, the means deployed by the French pilot have the primary objective of reducing consumption.

## Reducing consumption through technology

**Figure 3.**
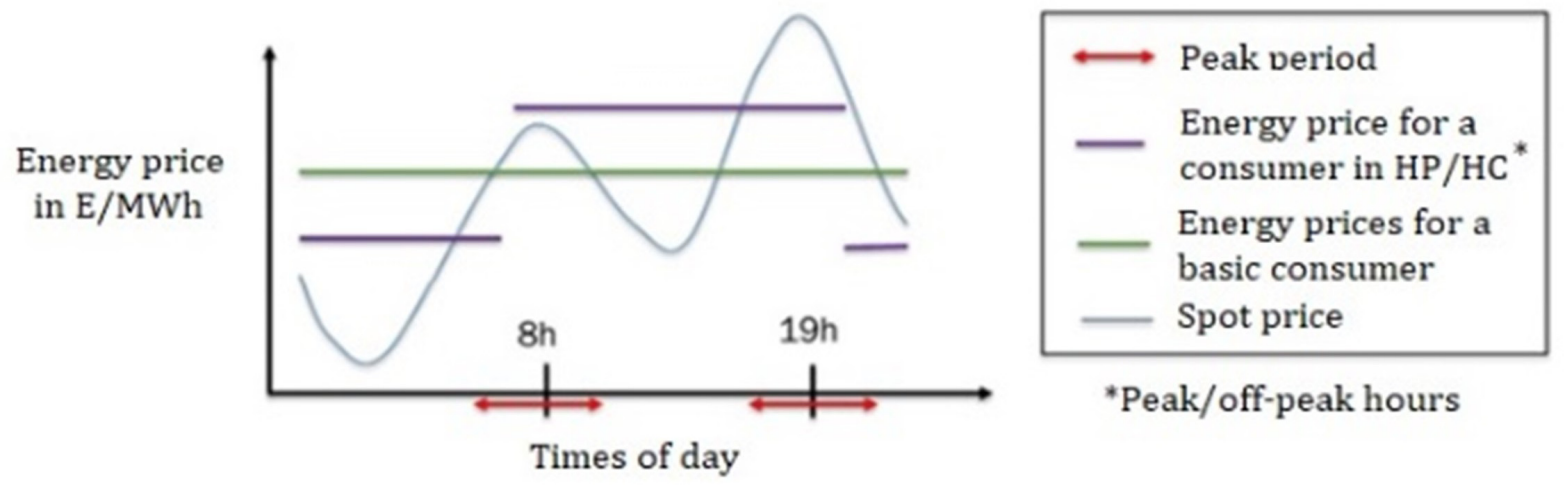
Example of qualitative variations in energy prices over the course of a day (source: ENGIE. Copyright permissions granted).

At a time of real tension in the energy market, InterConnect is trying to reduce end-user electricity consumption by 15% by relying on the dynamic tariff ^12^, ( Figure 3) which follows the movements of the energy market based on fluctuations between production and demand in Europe. The IOT systems (connected objects), sensors and control tools deployed by each pilot should help to achieve this goal. The general idea is not to make the devices concerned more energy-efficient, but rather to rationalise their use so that they provide the same service but optimise the times of consumption, if possible outside the hours of high voltage on the distribution networks, by triggering their operation at the right moment.

For example, turning on electric radiators just a few minutes before the occupants of a room arrive, rather than maintaining the desired temperature over time or turning them on at maximum power at the last moment, generates obvious savings.

In addition, the use of the latest generation of connected household appliances further enhances the efficiency of the project. These rather favourable energy class means, switched on at the most advantageous times of the day by remote triggering, also generate substantial savings either by reducing consumption or by choosing the least expensive time.

Let's take the example of a household with solar panels, during the time of day the energy cost is the most advantageous. The machine can be switched on when the production is the most interesting (high price and high self-generation) and in case of bad weather forecast or sudden deterioration of the sunshine conditions, the user can choose to switch on the machine in the least expensive time slot. Simple programming does not allow such flexibility.

It is also important to note that most of the appliances installed in the project no longer have belt-driven drums, but work by induction, which results in even greater energy savings.

## From experimentation to pedagogy

According to Hoffman & Novak (2018), "The Internet of Things (IoT) has the potential to revolutionise the consumer experience" in that the new usage experiences they offer, have the potential to revolutionise consumers' daily lives. Moreover, their penetration into the home shows that their appropriation contributes to the creation of value by consumers.

The initial feedback (from the first unpublished responses to the questionnaires of the quantitative study - 49 questionnaires) from this vast experiment deployed in seven European countries and involving 3,500 testers highlights the educational virtues of such a project. Indeed, whatever the testers, if the idea of making energy savings is the primary reason for participating in the experiment, the fact that they can contribute to generating additional savings by being involved in their own consumption encourages participants to get involved over the long term.

**Figure 4.**
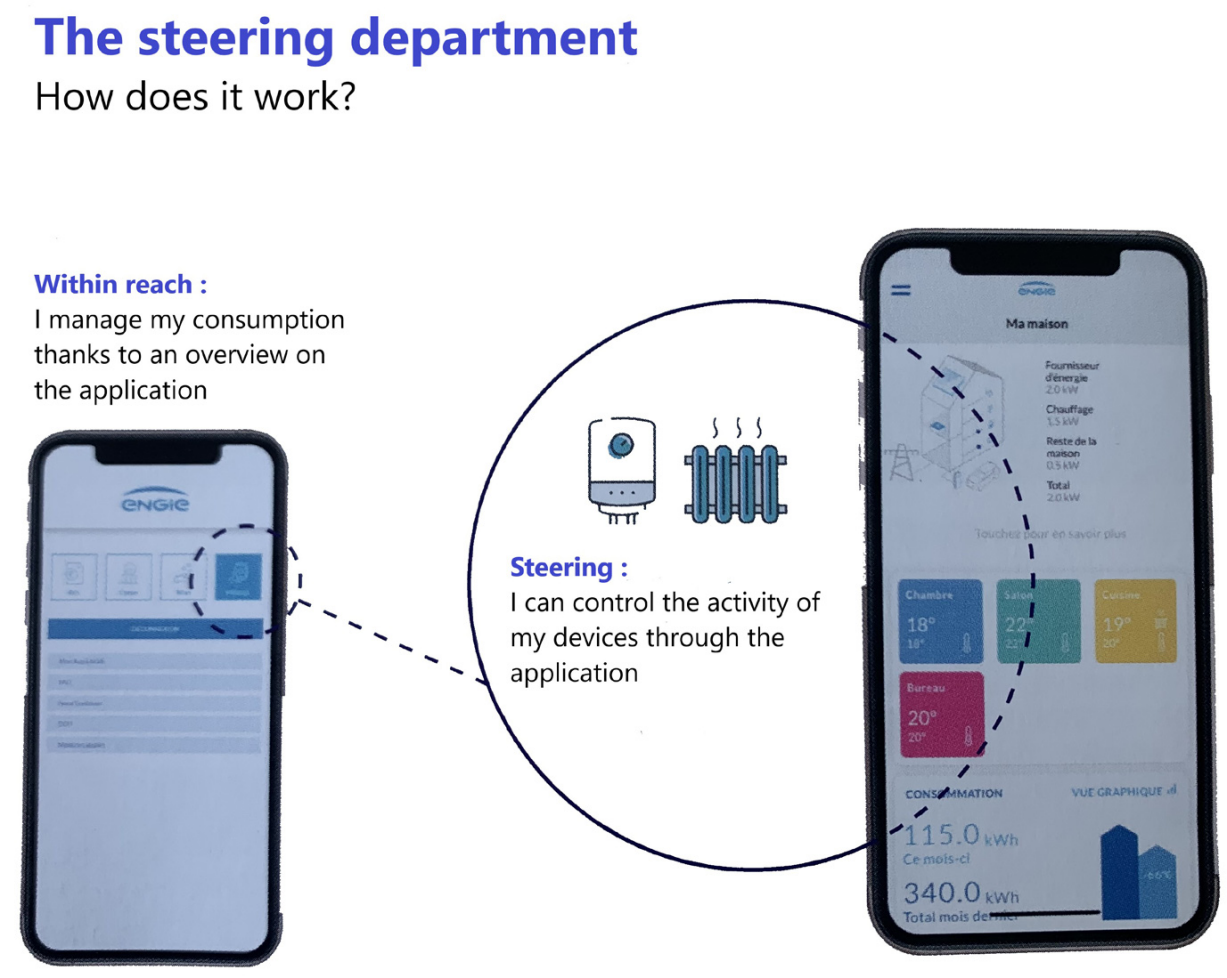
ENGIE Mobile Appliance (source: InterConnect presentation leaflet for ENGIE. Copyright permissions granted).

The mobile application ( Figure 4) that accompanies the remote monitoring and control equipment plays an essential role for users. It allows them to visualise in real time the savings made. This application is part of the services offered by ENGIE to its customers and has been adapted to take into account the sensors deployed as part of the InterConnect project. The simplicity of the principle encourages everyone to do better the next day. This playful aspect, rewarded by an almost instantaneous gain, plays an important educational role.

Moreover, thanks to this tool, the tester becomes a bit of a co-constructor of his or her usage. Usage, which refers to the notion of habit, is understood as a "social construct" that involves various levels, namely "the genealogy of usage, the appropriation process, the development of the social link, and the integration of usage into social relationships" (Jouët, 2000). The common four-stage methodology (Knowledge - Definition - Demonstration - Evaluation) adopted by each pilot of the InterConnect project is entirely consistent with the first two levels proposed by Josiane Jouët. For his part, the user, thus involved, can also improve the energy efficiency of his installation by installing new connected objects (switches, light bulbs, small household appliances, etc.).

Gradually, the user "develops habits" and says to themselves that it would be a shame to "waste" the potential gains of the experiment. It is obvious, despite the little hindsight we have on this experiment, that the educational virtue of the mobile application will allow for lasting changes in the behaviour of these consumers. First of all, on an individual basis, by motivating them to generate more savings and by the progressive awareness that is created. Then, quickly, this change in individual behaviour spreads to the immediate environment and neighbourhood. Either by encouraging people to join the experimentation or by conveying a positive message about these technologies. This "oil spot" effect is difficult to measure but remains a strong expectation of the project. It is important to remember that social uses are generated by individuals or groups of individuals. Serge Proulx emphasises that with the development of web technologies, we are witnessing "collective and networked uses" ( Proulx) of new technologies. In the context that interests us, in order to further stimulate users, the creation of a blog for them contributes to this sharing.

**Figure 5.**
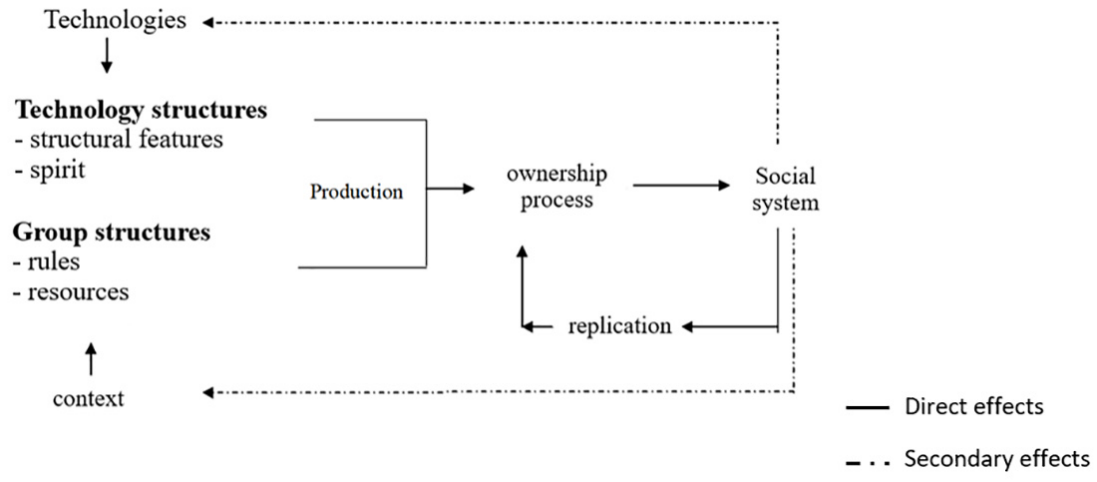
From use to practice: appropriation processes.

The following diagram ( Figure 5), inspired by the work of Poole and DeSanctis (1990) process of technology appropriation in the organisational context and adapted by Angélique Roux ( Roux, 2007), highlights this dynamic aspect of the appropriation process.

In order to measure these changes in behaviour, a study of societal impacts is being carried out throughout the project.

The quantitative part of this study consists of three questionnaires (not yet published) are to be distributed at the beginning, middle and end of the experiment in order to take stock of the situation before the project, to observe the first impacts at 12 months and the lasting impacts at the end of the project. These questionnaires cover 4 items, namely

- adaptation of premises and equipment
- energy management
- changes in comfort
- savings made.

The qualitative part of the study will be based on the semantic study of the interview reports of a representative panel of 6 to 7 end users of each pilot.

## Energy communities, win-win projects

In May 2019, the European Union adopted the Clean Energy Package ^13^. The third priority of this initiative is to put the consumer at the heart of the energy system. To this end, the EU asks Member States to support the creation of energy communities by promoting the two concepts of renewable energy communities and citizen energy communities.

The awareness and sensitisation created by the InterConnect project among user groups creates the right conditions to push experimentation further by encouraging them to form energy communities. In this win-win system, our "experimenters/consumers" become real experts in better consumption.

Some pilots already rely on existing energy communities to conduct their experimentation. Others, like the French pilot, have set themselves the objective of taking advantage of the positive effects of the experiment to organise one or more energy communities.

The idea of the French pilot is to support a municipality, a condominium or a group of consumers in their efforts to create an energy community using the tools developed within the framework of InterConnect, such as the "smart orchestrator" platform. This community should be structured around two axes:

- That of energy production (collective or individual). In this case, the producers decide to supply their surplus production to the community. The Smart Orchestrator registers the production of each individual and redirects it in priority to the members of the community.
- The second axis is consumption. Community members prioritise the consumption of green energy by triggering their consumption when it is available.

Production and consumption, recorded by the smart meter, are then rewarded by a virtual currency ("green-coin") or an existing local currency to which the reward system would be linked, and would be used in local shops (short cycle), to pay for services (vehicle recharging) or to reinvest in improving production capacities.

This notion of rewarding a virtuous act, be it production or consumption, is certainly an essential element in the constitution of a community, in any case, this is what we will try to demonstrate in our experiment. This community must be perceived and experienced as a project of general interest that meets economic development objectives, biodiversity conservation objectives and social interest protection objectives. The "reward" must not only compensate, but also offset. Compensation would facilitate the social and environmental acceptability of such projects.

The ideal, but not exclusive, community model could be carried by a municipality that invests in renewable energy production (e.g. solar panels installed on municipal buildings) and organises a community of self-consuming homeowners and tenants of social landlords. In this case, social acceptability would be ensured by the participation of all types of taxpayers, environmental acceptability would be guaranteed by the type of production chosen collectively and the economic aspect rewarding both the consumer and the producer would make it possible to promote local exchanges, improve (investment) and maintain the communal renewable energy production system.

**Figure 6.**
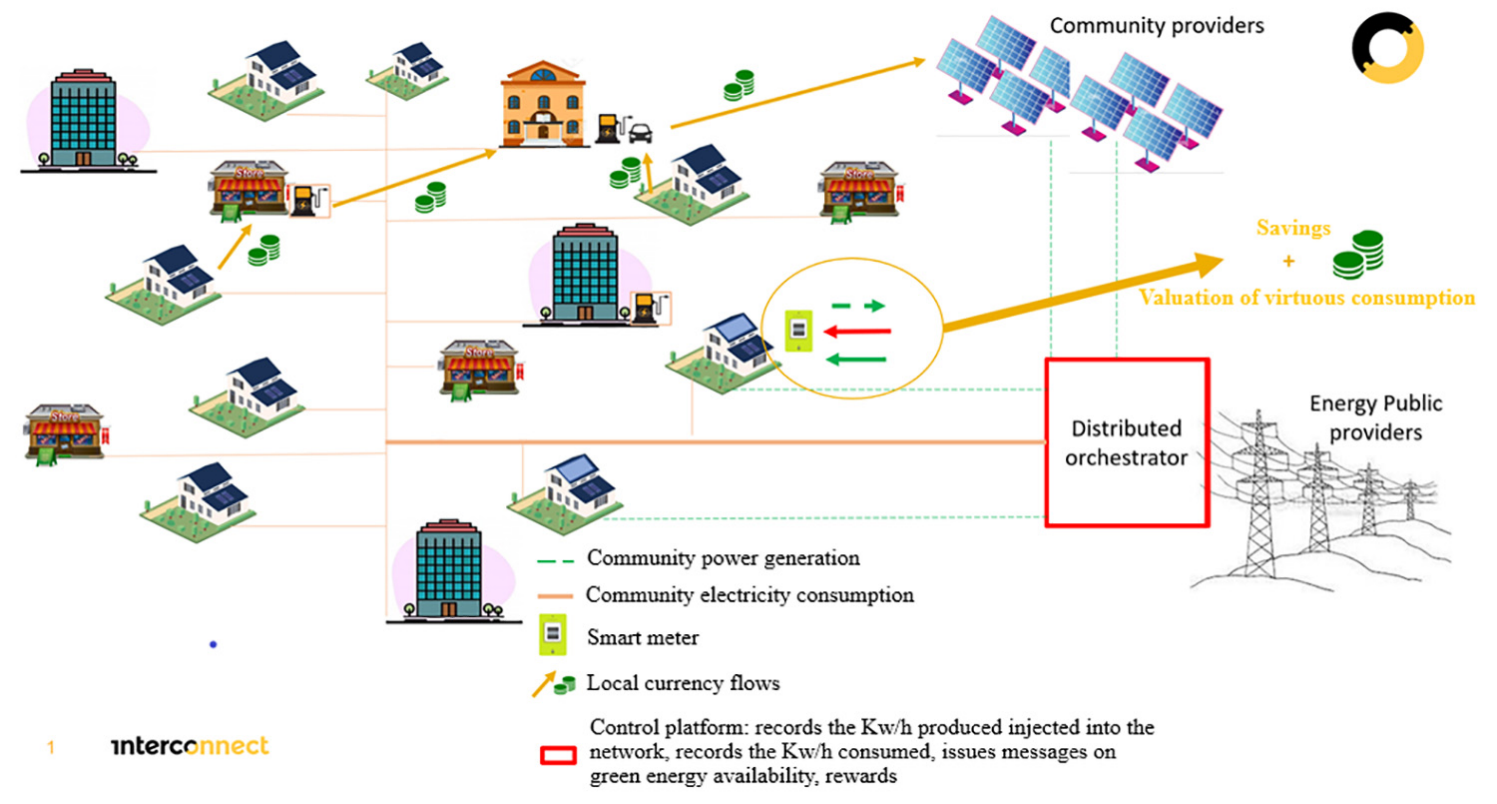
Energy community model for the InterConnect project pilot France.

**Figure 7.**
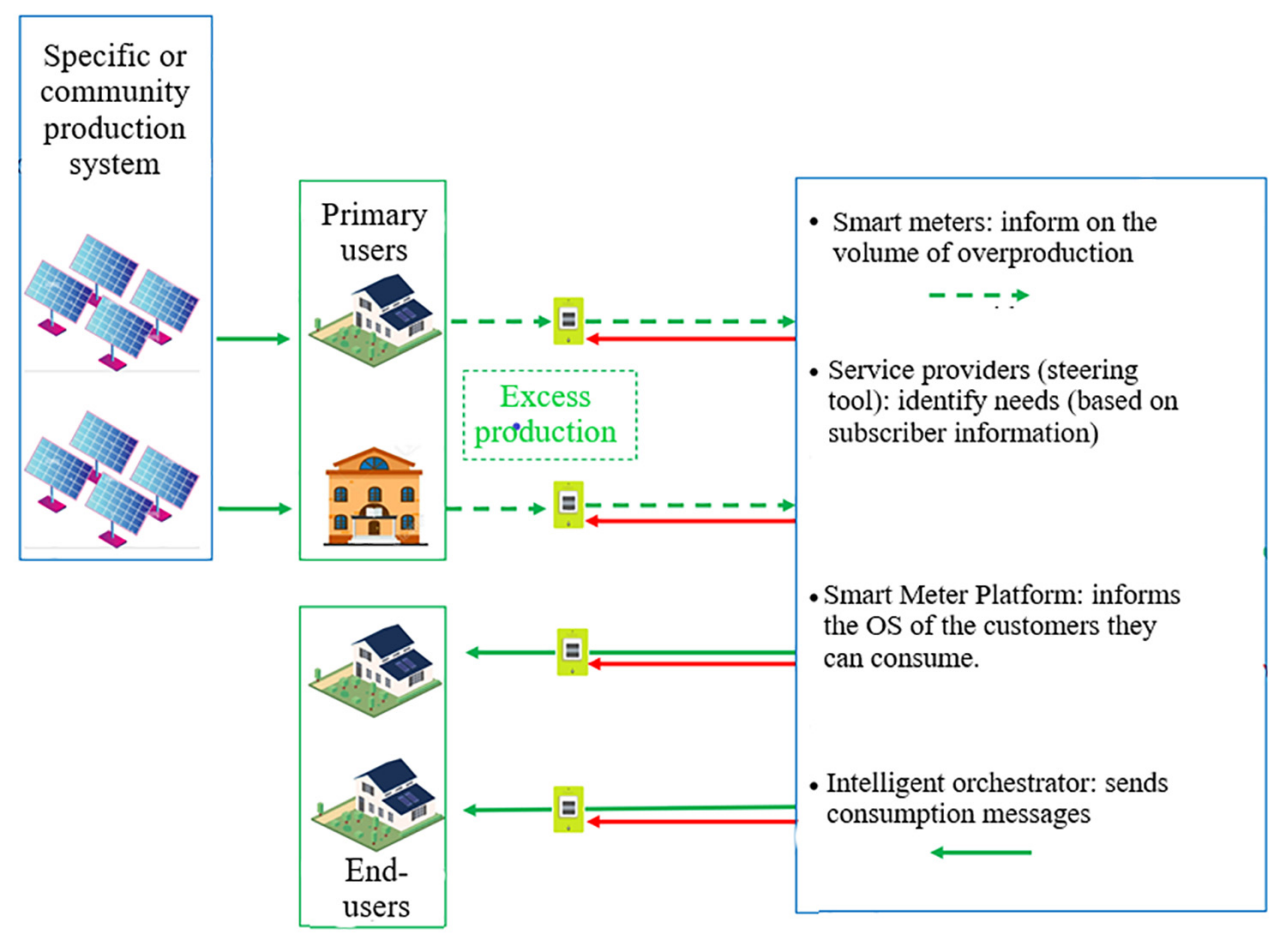
Additional aspects of energy community model for the InterConnect project pilot France.

The diagram ( Figure 6) details the principle of exchange with the network and within the community. The Smart Orchestrator platform (currently being developed for the project, not yet available) acts as a "switch". When a PV user does not need it, the surplus is made available to other users in the community ( Figure 7). Thus, it registers the input equivalent as green energy and informs the users of the most opportune moment to consume "green" and accounts for the consumption of each community member. These overproduction and green consumption are converted into points that allow the user to obtain discount vouchers from partner shops or to convert them into local currency to pay in partner shops or to recharge an electric vehicle.

The creation of one or more energy communities based on the InterConnect project experiments would have a particularly remarkable effect at this time of tension on energy prices, since it would combine resources designed to reduce electricity consumption by 15% with a capacity to produce renewable energy that could further reduce the need for the public grid.

**Figure 8.**
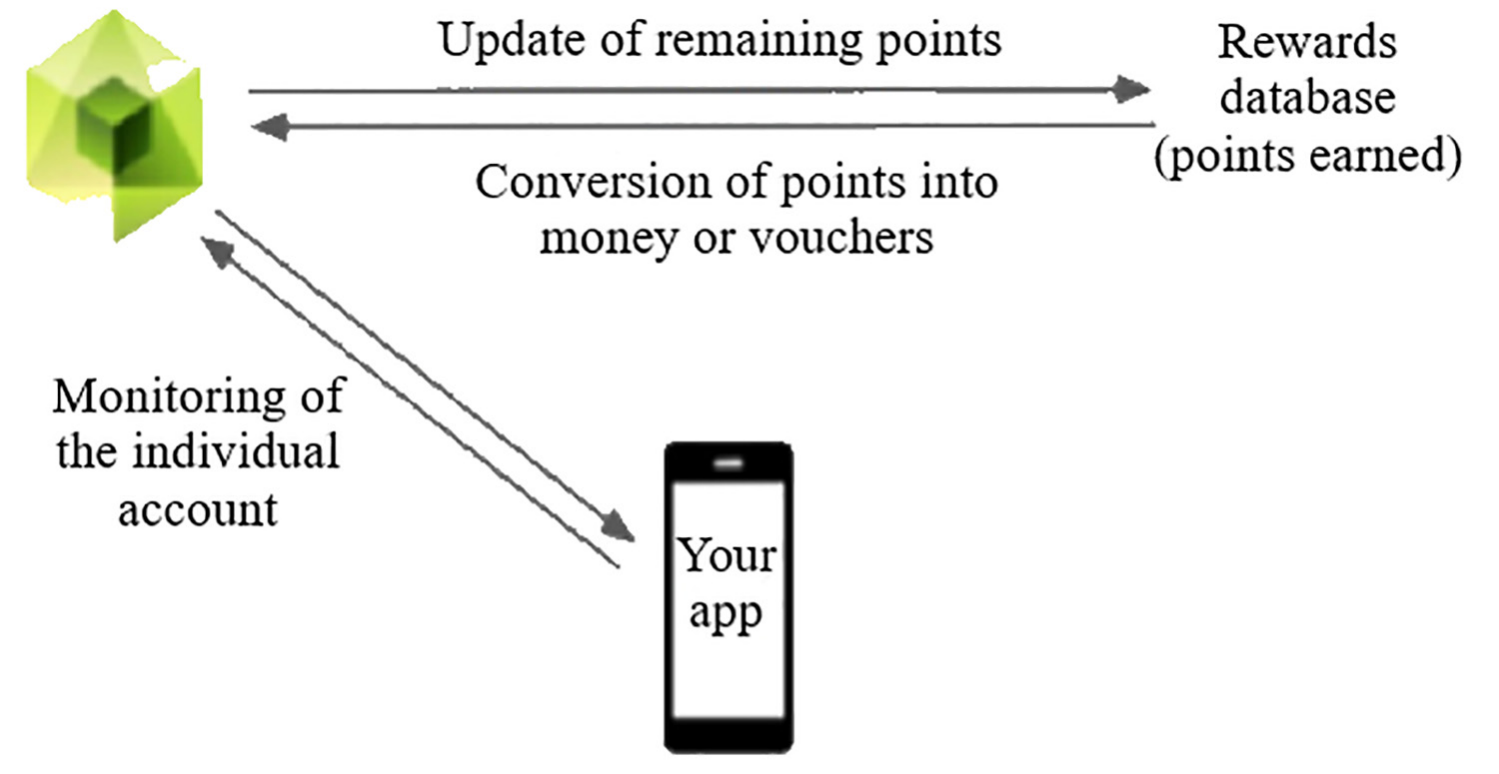
Principle of remuneration within the energy community.

The reward system is managed by an independent operator who is responsible for collecting discounts in the listed shops and converting the points obtained by users ( Figure 8).

## Conclusions

Because of the strong tensions on the energy market and the European Union's choice to deal with them in a community way, InterConnect is a project that is totally in line with current events. Through the volume of the experiments and the diversity of the means implemented, it is an opportunity to select pragmatic solutions that will make it possible to reduce the consumption of the most widespread equipment in European homes (electric heaters and electric water heaters).

The educational virtues of such an initiative based on a win-win principle, where energy suppliers are subject to less demand from a population that knows how to consume better and is aware of its essential role in the environment, is an encouraging first step towards achieving the ambitious EU objectives set out in the December 2015 Paris Agreement in the energy-climate frameworks for 2030 to reduce emissions by at least 40% compared to 1990.

Finally, this awareness-raising among consumers, making them true consumer actors in the energy market on an individual level, has led us to propose the basis of an energy community model that could become an effective solution for reducing the use of fossil fuels while democratising renewable energy. 

Because it fully meets the needs of the moment and the choices of the future in terms of energy consumption and management, the InterConnect project has a dual economic and educational interest among users, which should lead to responsible and sustainable behaviour.

The originality of this project therefore lies largely in the choice, not to create innovation for its own sake, but rather to create innovations that make the existing more modern and more economical. In a context of economic and social crises, this approach is bound to meet with a favourable response from the low-income customers, who are also the most numerous users of energy-consuming equipment.

In the 15 months or so before the end of the project (March 2024), it will be interesting to measure the societal impacts it has produced. An important semantic study is being conducted in this context and will lead to the identification of the most relevant vocabulary to be used to provide arguments to support future European projects or the marketing of solutions tested in InterConnect.

## Data Availability

No data are associated with this article.
