# Supplementary material for: InterConnect: A European project that is changing the way energy is consumed
Source: Open Res Eur. 2023 Mar 1;3:41. [Version 1] doi: 10.12688/openreseurope.15227.1 (PMC10445883; doi:10.12688/openreseurope.15227.1)
Supplement: Peer review history

# Peer review: Reviewer response for version 1

**DOI:** 10.21956/openreseurope.16469.r33671

**Published:** 2023-08-15

**Authors:** Shiyu Yang

**Affiliations:** 1 Cornell University, Ithaca, New York, USA

The paper presents the InterConnect project, which aims to reduce energy consumption and promote responsible and sustainable behavior among its users. The project's key innovation is its interoperability platform, which allows for the integration of a broad range of technologies and enables the configuration and management of microgrids, charging systems for electric vehicles, and technologies for controlling and managing connected objects. Although limited technical details are provided, the interoperability platform could be useful for improving the energy efficiency of the connected and complex existing systems in homes and buildings. The project also focuses on raising awareness and educating users about energy consumption and management through its mobile application and energy community model. Scale-up of the solutions in the project may face some social and technical challenges, which are also worth discussing.

Where applicable, are recommendations and next steps explained clearly for others to follow? (Please consider whether others in the research community would be able to implement guidelines or recommendations and/or constructively engage in the debate)

Partly

Does the article adequately reference differing views and opinions?

Yes

Are all factual statements correct, and are statements and arguments made adequately supported by citations?

Yes

Is the rationale for the Open Letter provided in sufficient detail? (Please consider whether existing challenges in the field are outlined clearly and whether the purpose of the letter is explained)

Partly

Is the Open Letter written in accessible language? (Please consider whether all subject-specific terms, concepts and abbreviations are explained)

Yes

Reviewer Expertise:

Intelligent building control, Building decarbonization, Building energy efficiency, Renewable energy integration

I confirm that I have read this submission and believe that I have an appropriate level of expertise to confirm that it is of an acceptable scientific standard.

# Peer review: Reviewer response for version 1

**DOI:** 10.21956/openreseurope.16469.r33674

**Published:** 2023-07-31

**Authors:** Sumit Rathor

**Affiliations:** 1 Department of Electrical Engg and Computer Science, Khalifa University, Abu Dhabi, Abu Dhabi, United Arab Emirates

The author presented the manuscript "InterConnect: A European project that is changing the way energy is consumed," aiming at minimizing households' energy consumption through experiments on 3500 users. The study relies on IoT applications to control household devices through signaling and educating users about the need to reduce energy usage. There are many such studies conducted by researchers through different simulation strategies however the presented study is at a demonstration level, providing an excellent contribution to the research community. Still, there is scope for studying users' comfort levels by adjusting their devices based on power demand curves.

Where applicable, are recommendations and next steps explained clearly for others to follow? (Please consider whether others in the research community would be able to implement guidelines or recommendations and/or constructively engage in the debate)

Yes

Does the article adequately reference differing views and opinions?

Partly

Are all factual statements correct, and are statements and arguments made adequately supported by citations?

Yes

Is the rationale for the Open Letter provided in sufficient detail? (Please consider whether existing challenges in the field are outlined clearly and whether the purpose of the letter is explained)

Yes

Is the Open Letter written in accessible language? (Please consider whether all subject-specific terms, concepts and abbreviations are explained)

Yes

Reviewer Expertise:

Energy, Policy, Electric Power System, Renewable Energy Source Integration

I confirm that I have read this submission and believe that I have an appropriate level of expertise to confirm that it is of an acceptable scientific standard.

# Peer review: Reviewer response for version 1

**DOI:** 10.21956/openreseurope.16469.r31979

**Published:** 2023-06-13

**Authors:** Nebojša Jurišević

**Affiliations:** 1 University of Kragujevac, Jovana Cvijića, Kragujevac, Serbia

The study InterConnect: A European Project Changing the Way Energy Is Consumed describes the current status of a project aimed at improving energy management in societies by focusing on energy consumption in buildings. In this regard, the study presents the findings of a pilot project in France, one of seven countries included in InterConect. In particular, the study is based on IoT applications in 1) residential and 2) public buildings. The study aim is to manage building electricity demand so that energy is not only used more efficiently, but also synchronized with fluctuations in electricity generation and demand. This should be made possible by adopting a dynamic electricity tariff, which would cause electricity prices to fluctuate in relatively discrete time intervals. Using this solution would optimize the “energy behavior” of the buildings more than the relatively simple programming available in non-dynamic tariff systems.

 Although some of the data mentioned in the study are still unavailable, the study makes a significant contribution to the professional and general public by highlighting all of the challenges research team had to overcome in order to properly deploy and investigate the solution effectiveness.

 Aside from demonstrating direct benefits from technology use, the paper also confirms the indirect effect through citizen education and the formation of energy communities. The findings presented in this paper can be used as examples of good practice to inspire similar actions in other regions.

Where applicable, are recommendations and next steps explained clearly for others to follow? (Please consider whether others in the research community would be able to implement guidelines or recommendations and/or constructively engage in the debate)

Yes

Does the article adequately reference differing views and opinions?

Yes

Are all factual statements correct, and are statements and arguments made adequately supported by citations?

Yes

Is the rationale for the Open Letter provided in sufficient detail? (Please consider whether existing challenges in the field are outlined clearly and whether the purpose of the letter is explained)

Yes

Is the Open Letter written in accessible language? (Please consider whether all subject-specific terms, concepts and abbreviations are explained)

Yes

Reviewer Expertise:

Energy Efficiency, Energy Management, Energy and Buildings

I confirm that I have read this submission and believe that I have an appropriate level of expertise to confirm that it is of an acceptable scientific standard.
